# Rapid Kinetic Fluorogenic Quantification of Malondialdehyde in Ground Beef

**DOI:** 10.3390/foods14142525

**Published:** 2025-07-18

**Authors:** Keshav Raj Bhandari, Max Wamsley, Bindu Nanduri, Willard E. Collier, Dongmao Zhang

**Affiliations:** 1 Department of Chemistry, Mississippi State University, Starkville, MS 39762, USA; krb663@msstate.edu (K.R.B.); mw2156@msstate.edu (M.W.); 2 Department of Basic sciences, Mississippi State University, Starkville, MS 39762, USA; bnanduri@cvm.msstate.edu; 3 Department of Chemistry, Tuskegee University, Tuskegee, AL 36088, USA

**Keywords:** malondialdehyde, lipid oxidation, ground beef, fluorogenic, kinetic fluorescence

## Abstract

Malondialdehyde (MDA), a mutagenic and carcinogenic compound, is widely studied in the meat industry and lipid peroxidation research due to its implications for food quality and safety. Current methods for quantifying MDA in solid tissues are labor-intensive, requiring multiple instruments and approximately two hours to complete. This study presents an ultrafast kinetic fluorogenic method for quantifying MDA in ground beef, utilizing 2-thiobarbituric acid (TBA) as a fluorogenic probe. The total assay time is significantly shortened to 6 min from sample preparation to data acquisition. The assay’s robustness against matrix interference was validated using sample volume variation and standard addition calibration methods. Additionally, the effects of ambient exposure to air, washing, and cooking on MDA content in raw ground beef were quantified. While both ambient exposure to air and cooking increased MDA levels, washing raw ground beef and decanting cooked ground beef broth effectively reduced MDA levels in the ground beef. This simple and rapid assay can be adopted both in food research and industry. Moreover, insights from our study on the relationship between ground beef treatment and MDA concentration will help consumers make informed decisions about ground beef handling and consumption to lower their intake of MDA.

## 1. Introduction

Malondialdehyde (MDA), a three-carbon dialdehyde, is a widely recognized biomarker for lipid peroxidation and the degradation of meat and meat products [1,2,3]. It forms during the oxidative breakdown of polyunsaturated fatty acids [4], resulting in off-flavors [5,6], diminished nutritional value [7,8], and potential health risks from the production of reactive carbonyl compounds [9,10]. Studies have indicated that MDA can form adducts with DNA, leading to mutations that might contribute to carcinogenesis [11,12]. Therefore, minimizing MDA formation is important for disease prevention. Accurate and rapid quantification of MDA in meat is crucial for evaluating its quality, shelf-life, and safety. Knowledge about MDA content in meat is important for consumers to make the best decisions about meat handling and consumption.

Current MDA quantification is mostly based on optical spectroscopic methods using the well-known condensation reaction between MDA and 2-thiobarbituric acid (TBA). MDA is optically transparent in the UV-vis region, but the TBA–MDA adduct, produced by the TBA and MDA reaction, is both chromogenic (CG) and fluorogenic (FG). The amount of MDA can be quantified using either UV-vis or fluorescence signals of TBA-treated sample solutions. Among the various UV-vis and fluorescence-based MDA assays, the simplest method involves determining the MDA concentration after measuring the UV-vis intensity at 532 nm—the characteristic absorption peak of the TBA–MDA adduct—following TBA’s reaction with the sample extract. While this approach is simple to perform and compatible with high-throughput assays using microplate readers, it suffers from two drawbacks: poor selectivity and low assay efficiency (60–90 min assay time).

This poor selectivity is due to TBA being broadly reactive to aldehydes and some other species (proteins, pyrimidines, etc.) that might be present in sample extracts. These reaction products can also absorb at 532 nm [13], making the MDA concentration falsely high. As such, most reports refer to this method as an assay of TBA-reactive substances (TBARS). While TBARS assays have been very popular in biological research [14], care must be exercised in the interpretation of the TBARS level because the signal response of the different TBARS can differ significantly. The TBARS assay is broadly recognized as the benchmark for MDA quantification, thanks to its simplicity, low cost, and longstanding application in lipid oxidation research [15]. Though it lacks perfect specificity, its versatile colorimetric or fluorometric detection and suitability for a wide range of sample types maintain its widespread use.

Post-reaction sample separation, such as high-performance liquid chromatography (HPLC), has been used for improving the assay’s selectivity. In this case, the MDA content is quantified based on the UV-vis or fluorescence signal of the fraction that contains TBA–MDA adduct. Although this approach ensures selectivity for the TBA–MDA detection, it is considerably more expensive due to its lengthy, labor-intensive procedures and the demanding instrument requirements.

In addition to UV-vis and fluorescence assays, surface-enhanced Raman spectroscopic (SERS) methods have also been used for TBA–MDA quantification by taking advantage of the excellent TBA–MDA SERS activities [16]. Regardless of the specific method of detection, the current UV-vis-, fluorescence-, and SERS-based MDA assays are all equilibrium spectroscopic quantification, i.e., the MDA content is determined after completion of the TBA–MDA reaction (60–90 min).

Recently, we reported the theoretical model for a kinetic fluorogenic (FG) MDA assay and applied it to quantify MDA content in chicken meat based on the fluorescence intensity change during the TBA–MDA reaction. The kinetic fluorescence MDA quantification is based on the slope *m*_*x*_(*T*) in the linear time course region of the kinetic fluorescence obtained in the TBA/MDA reaction solution. Mathematically, this slope is linearly proportional to the MDA concentration (Equation (1)),

(1) $$m_{x}(T)=k(T)C_{x}$$

A detailed derivation of the method has been presented in a previous publication and is referenced herein for completeness [17].

The key advantage of kinetic FG quantification is its assay speed (4–6 min) because it deduces the analyte concentration based on measurements taken during the reaction, not after the reaction, as in equilibrium quantification. While kinetic spectroscopic quantifications were proposed more than half a century ago [18], their applications have been limited mostly to research samples with no matrix complications [19,20]. To be widely adopted, kinetic spectroscopic quantification must be capable of mitigating matrix interference through strategies that include careful pre-reaction sample preparation, reaction design, and spectroscopic measurements.

The methods to mitigate matrix interference in our kinetic FG TBA-based MDA assay include large sample dilution, large TBA excess, and careful selection of the fluorescence excitation and detection wavelengths. Sample dilution with large TBA excess not only ensures the validity of the two sequential pseudo-first-order reactions model used for data analysis, but it also reduces the impact of interfering reactions of TBA with non-MDA TBARS or MDA reactions with matrix species. Further, sample dilution also reduces interference of matrix species that can affect reaction kinetics of TBA and MDA or the optical properties of the TBA–MDA adduct.

The current study is a critical extension of our kinetic FG MDA quantification method with four key objectives. First, to further improve the assay’s sensitivity and efficiency. One key finding is that a (1:1, *v*:*v*) dimethyl sulfoxide (DMSO) and water solvent offers an optimal combination of sensitivity and efficiency. Second, to broaden the applicability of this kinetic FG MDA method. We successfully quantified MDA in ground beef, a red meat that presents more complex matrix interferences than chicken, a white meat used in our proof-of-concept study. Third, to conduct a head-to-head comparison of the reliability of kinetic and equilibrium-based FG assays. One important insight is that kinetic FG quantification can be significantly more reproducible than equilibrium FG assays because it is less affected by instrument and sample background fluorescence, making it a more reliable method. Fourth, to evaluate ground beef handling and treatment on MDA concentration, including the determination of the MDA concentration in the beef broth and solid residue of cooked ground beef. We found that MDA concentration is significantly higher in the ground beef broth than the cooked ground beef. Such information can be important for consumers to make informed decisions regarding beef preparation and consumption.

## 2. Materials and Methods

### 2.1. Materials

All chemicals, unless specified otherwise, were purchased from Sigma Aldrich (St. Louis, MO, USA) and used without further modification. Nanopure water (18.2 MΩ cm^−1^, Thermo Scientific, Waltham, MA, USA) was used throughout and is referred to as water. Fluorescence spectra were acquired with a FluorMax-4 spectrophotometer (Horiba Jobin Yvon, Edison, NJ, USA) equipped with a Quantum Northeast Luma 40/Horiba4 temperature controller. Steady-state and kinetic UV-vis spectra were acquired with a Shimadzu UV-2600i UV-vis Spectrophotometer (Duisburg, Germany) equipped with a Shimadzu CPS-100 temperature controller. The accuracy of the temperature controllers is ±0.1 °C for both instruments. A Magic Bullet blender (250 W motor, high-torque power base) of speed 10,000 rpm was used for blending all samples, and a stainless-steel tea filter (6″ L × 3″ W × 1″ H) with a pore size of 100 µm was used to separate solids from the liquid. The liquid was then filtered using 0.2 µm PES syringe filters (VWR) attached to 10 mL syringes. All experiments were conducted in triplicate (*n* = 3).

### 2.2. Preparation of MDA and TBA Solutions

A 20 mM solution of MDA was prepared by mixing 20 μL of tetraethoxypropane (TEP) with 6 mL of 1% H_2_SO_4_ [17,21]. A stoichiometric amount of MDA was generated by leaving the solution at room temperature for 2 h [16,22]. A 205.2 mM TBA solution was prepared by dissolving 0.5915 g of TBA in 20 mL of a 1:1 volume mixture of DMSO and water. To facilitate TBA dissolution, the solution was heated to 60 °C. A 500 µL aliquot of 1.3 mM aqueous phosphoric acid was added to 2.42 mL of a 205.2 mM TBA solution.

### 2.3. Preparation of TBA–MDA Adduct

In a round-bottom flask, 0.6521 g of TBA was dissolved in 100 mL of water, followed by the addition of 100 µL of 20 mM MDA stock solution. The mixture was heated at 90 °C for 120 min. After cooling the reaction mixture for one hour, small crystals of TBA–MDA adduct began to form. The resulting crystals were then filtered, washed with water, and allowed to air dry under ambient conditions for 24 h. 10 mM stock solutions of the TBA–MDA adduct were prepared in both water and DMSO by dissolving 64.60 mg of the adduct in 20 mL of each solvent. The solutions were diluted in their respective solvents to achieve a final concentration of 1 µM. A series of 1 µM TBA–MDA adduct solutions of 99%, 90%, 80%, 75%, 60%, 50%, 40%, and 25% DMSO were prepared by mixing the appropriate volumes of 1 µM TBA–MDA adduct DMSO and water solutions. These TBA–MDA solutions were used in solvent-dependent studies.

### 2.4. Preparation of Extracts of Ground Beef

Approximately 25 g of ground beef (73% lean and 27% fat) was obtained from ground beef purchased at a local Kroger in Starkville, MS, USA (Kroger^®^ 73/27 Ground Beef Tray 3 LB, UPC: 0001111097568). A single batch was used throughout the experiment to minimize variability in fat and protein content across replicates (*n* = 3). Each sample was blended with 100 mL of water for 30 s. After blending, the solid portion was removed, and the liquid fraction was filtered to obtain the filtrate, referred to as the extract of ground beef, then 1 mL of filtrate was taken for analysis.

### 2.5. Sample Volume Variation

In the sample volume variation experiment, four volumes (40, 80, 100, and 120 µL) were taken from the freshly prepared extract of ground beef prepared as described in Section 2.4. Each volume was then added to a separate solution containing 2.42 mL of 205.2 mM TBA and 0.5 mL of 1.3 M phosphoric acid.

### 2.6. Preparation of Sample for Standard Addition Method

To validate our kinetic method, varying concentrations of malondialdehyde (MDA) were added to a series of 600 µL extracts of ground beef. Specifically, MDA was introduced at concentrations of 0, 20, 40, 80, and 120 nM, corresponding to volumes of 0, 40, 80, 160, and 240 µL, respectively. Each solution was then brought to a final volume of 1 mL with the addition of 1% sulfuric acid. The extract of ground beef was prepared following the procedure outlined in Section 2.4.

### 2.7. Preparation of Extracts of Ground Beef Exposed to Air

Six samples (*n* = 3), each containing 25 g of ground beef from the same batch, were placed individually in plastic containers (14 cm × 14 cm × 5 cm), and each ground beef sample was spread evenly on the bottom of its container. The containers were then placed on a laboratory bench top under ambient conditions for intervals of 0, 1, 3, 6, 16, and 24 h, respectively. After each interval, the exposed ground beef samples were blended with 100 mL of water and then filtered to obtain extracts of ground beef exposed to air. From each filtrate, 1 mL was taken for analysis.

### 2.8. Preparation of the Extracts of Washed Ground Beef

Five samples (*n* = 3), each containing 25 g of ground beef, were soaked in 100 mL of water with constant stirring for durations of 0, 1, 2, 3, and 4 min, respectively. From each sample, water was decanted, and the remaining ground beef was blended with 100 mL of water, followed by filtration to get extracts of washed ground beef. After filtration, 1 mL of each sample was retained for analysis.

### 2.9. Preparation of Extracts of the Cooked Ground Beef

A 25-g sample of ground beef (*n* = 3) was blended with 100 mL of water for 30 s. 1 mL of the mixture was taken and filtered to obtain the extract, which is referred to as the extract of raw ground beef. The remaining mixture was then cooked at 100 °C for 1 h with constant stirring. After filtration, 1 mL of the liquid portion, referred to as the beef broth, was used for MDA quantification. The remaining solid ground beef was blended with another 100 mL of water and then filtered to obtain the filtrate, called cooked beef. 1 mL of this cooked beef filtrate was used for the quantification of MDA.

### 2.10. Fluorescence Measurements

Steady-state and kinetic fluorescence measurements of the TBA–MDA reaction solutions were acquired using a procedure modified from the literature [23]. The phosphoric acid-containing TBA stock solution was placed in an oven maintained at 60 °C before analysis. The stock solution was preheated in an oven at 60 °C for 10 min to ensure uniform thermal equilibration before injection of the analyte solution. 2.42 mL of the preheated TBA stock solution and 0.5 mL of 1.3 M phosphoric acid were then pipetted into a 3.5 mL, 1 cm × 1 cm quartz cuvette preheated to 60 °C in the temperature-controlled cuvette holder. The 80 µL of MDA-containing solution was then added to the cuvette. Kinetic fluorescence spectra were acquired immediately upon the addition of MDA. Kinetic fluorescence measurements were taken with an excitation wavelength and slit-width of 532 nm and 3 nm, respectively, and a detection wavelength and slit-width of 567 nm and 28 nm, respectively. The integration time for each data point is 0.8 s, with an acquisition time interval of 1.0 s. A slit width of 3 nm was selected for excitation to target the specific wavelength region where the TBA–MDA adduct exhibits strong absorbance while minimizing background absorption from the sample matrix. An emission slit width of 28 nm was used to maximize signal collection from the TBA–MDA fluorescence and to reduce the impact of potential matrix interferences, ensuring high sensitivity and reliable detection.

### 2.11. Savitsky-Golay Derivatization

The identification of the linear time course region in each kinetic fluorogenic measurement was achieved using the second-order Savitzky–Golay digital derivatization with a window size of 11 points implemented using a Python 3.11.5 program and a graphical user interface (Text S1). This window size was specifically chosen to minimize noise interference, accurately capture rapid changes in signal slope, and ensure consistency across all measurements [24,25] (Figure S2). The linear time course region of the kinetic spectral intensity includes the temporal points where the first derivatives are no more than 5% below the maximum derivative.

### 2.12. Analytical Characteristics

The performance of the kinetic and equilibrium fluorescence methods was comprehensively evaluated by examining a series of critical validation parameters. Calibration curves were constructed by plotting the analyte concentrations (x, in nM) against their corresponding analytical responses (y), then generating the best fit linear equation,

y_i_ = mx_i_ + b

The limit of detection (LOD) and limit of quantification (LOQ) were determined through the simultaneous analysis of seven replicate samples. The limits of detection (LOD) and quantification (LOQ) were calculated using the equations:

LOD = 3 SD/m and LOQ = 3 LOD,

respectively, where x represents the analyte concentration of the standard calibration solution, m is the slope of the calibration curve, and SD is the signal standard deviation of the blank. To assess the method’s precision, the relative standard deviation (RSD%) was calculated for intra-day precision, based on three replicates analyzed within a single day, and inter-day precision, using three replicates analyzed across three different days. Recovery tests were conducted on samples that had been spiked with MDA.

### 2.13. Statistical Method

The analytical data were carried out in triplicate (*n* = 3), except for the statistical tests of significant difference (*n* = 7), and analyzed by OriginPro (2024). The sample results were presented as the mean ± standard deviation, reflecting both the central value and the variability of the data. Measurement precision across runs was evaluated and verified through the calculation of the relative standard deviation (RSD%). A paired *t*-test was performed to compare the mean MDA concentrations between equilibrium and kinetic methods.

## 3. Results and Discussion

### 3.1. Solvent and Temperature Effects on FG MDA Quantification

Most existing MDA assays, including the one previously reported by us, are performed using water as the reaction and/or detection solvent [17,21]. MDA quantification using water is more efficient with the kinetic method compared to the equilibrium method. For instance, MDA can be quantified in just 6 min using kinetic assays [17], whereas the equilibrium method requires at least 60 to 90 min [14,22]. However, efficiency is not the only factor in evaluating an assay; sensitivity is also a crucial factor. Given the poor solubility of TBA in water and the reduced fluorescence activity of the TBA–MDA complex in aqueous solutions, it’s crucial to explore and employ alternate solvents to optimize the assay. The ideal solvent should be inert to both TBA and MDA, while also facilitating rapid reaction kinetics and ensuring high detection sensitivity.

We explored a range of solvents, including water, acetic acid, *n*-propanol, dimethylformamide (DMF), and dimethyl sulfoxide (DMSO). TBA showed good solubility in DMSO, acetic acid, *n*-propanol, and DMF. However, DMF was excluded because of its reactivity with TBA. Subsequently, we investigated the impact of DMSO, acetic acid, and *n*-propanol on the fluorescence quantification of the TBA–MDA reaction. DMSO was the first choice due to its significant enhancement of the fluorescence signal.

**Figure 1:**
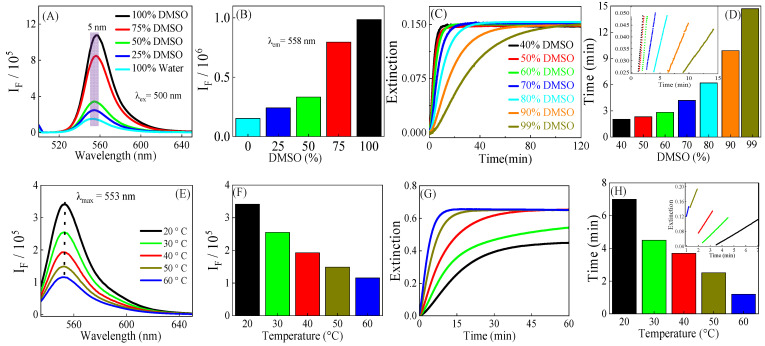
(**A**) The fluorescence spectra of 1 µM TBA–MDA in DMSO/water cosolvents of different DMSO volume fractions. (**B**) The fluorescence peak intensity as a function of DMSO volume fractions at λem = 558 nm. (**C**) The kinetic UV-vis of the reaction solutions of 1 µM MDA and TBA with DMSO/water cosolvents, with volume fractions shown in the legends. (**D**) The time interval to reach the end of the linear time course region as a function of DMSO volume fractions. Inset is the linear time course region. (**E**) The fluorescence spectra of 1 µM TBA–MDA in DMSO/water cosolvents at different temperatures are shown in the legend. (**F**) Fluorescence peak intensity as a function of temperature at λem = 553 nm. (**G**) The kinetic UV-vis of the reaction solutions of 1 µM MDA and TBA with DMSO/water cosolvents at different temperatures. (**H**) The time interval to reach the end of the linear time course region as a function of temperature. Inset is the linear time course region.

To investigate the influence of DMSO on the TBA–MDA reaction, a series of DMSO/water cosolvents with different volume ratios was prepared. The FG equilibrium assay showed that increasing the DMSO solvent fraction to 100% resulted in a 5 nm red shift of maximum peak emission (Figure 1A); more importantly, the fluorescence intensity had a drastic 550% increase when the DMSO volume fraction was increased to 100% compared to 100% water (Figure 1A,B). A similar observation was found in the CG equilibrium assay with a red-shifted maximum peak absorbance of 6 nm but only a 13% increase in UV-vis extinction spectra (Figure S3).

The kinetic CG spectra were obtained with the sample temperature maintained at 60 °C, as previously reported [17]. The kinetic CG spectra (Figure 1C,D) exhibited the characteristic patterns of a two-step reaction that is comprised of three distinct regions: an acceleration region, a linear time course region, and a deceleration region, which aligns with the literature [17]. The accelerating region is characterized by a rapid increase in spectral signals, while the decelerating region exhibits a gradual decrease in spectral signals. The linear time course region is defined as the time interval where the slope of kinetic data is within 5% difference from its maximum determined using the second order Savitzky–Golay derivative. The linear time course region is necessary for quantification of analytes because the change in the slope of kinetic signals in this region is linearly proportional to the concentration of the analyte. The endpoint of this linear time course region is used as the measurement time for determining the assay efficiency.

At constant TBA concentration, increasing the volume fraction of DMSO also reduces the reaction rate (Figure 1C,D), thereby decreasing assay efficiency. Consequently, the linear time course region for CG quantification exhibited a sharp exponential decline, resulting in a more than seven-fold decrease in assay efficiency. However, as the DMSO volume fraction increases, the solubility of TBA improves, making it more concentrated and readily available for reaction with MDA. This enhancement ultimately boosts assay efficiency. A 590 mM TBA solution was obtained in a 50% DMSO/water mixture, which is almost six times more concentrated than the 100 mM TBA solution in water. Utilizing this higher TBA concentration significantly improved the efficiency of the kinetic FG assay, reducing the reaction time from 6 min to just 3 min (Figure S4).

The data clearly reveal the impact of the solvent on the specific assay method employed. For CG quantification, a higher water solvent fraction is optimal because it offers a notable reduction in assay time by decreasing the end point of the linear time course region from 15 min to 2.5 min for DMSO solvent fractions of 99% DMSO and 50% respectively (Figure 1D). For high FG quantification sensitivity, however, the optimal detection solvent is DMSO, as the TBA–MDA adduct has its highest fluorescence intensities compared to other solvents evaluated. However, the reaction rate of TBA–MDA is very slow in DMSO, which is detrimental for assay efficiency. The DMSO/water cosolvent’s composition in the fluorogenic MDA assay revealed a tradeoff between sensitivity and efficiency; higher water content improves assay efficiency but decreases sensitivity, whereas increasing DMSO content enhances sensitivity at the expense of efficiency. The data indicated that (1:1, *v*/*v*) DMSO/water cosolvent is the optimal solvent in terms of assay efficiency and sensitivity.

After investigating the impact of solvents, we next explored how temperature influences assay efficiency and sensitivity (Figure 1E–H). Generally, increasing the reaction temperature reduces the reaction time, thereby improving assay efficiency. Our prior studies demonstrated that increasing the temperature significantly enhances the rate of the TBA–MDA reaction [17]. Unfortunately, elevating the temperature of the DMSO/water cosolvent above 60 °C led to significant bubble formation, which adversely affected the fluorescence measurements. Thus, for all kinetic fluorescence experiments, the highest temperature was 60 °C.

We also investigated how the fluorescence activity of the TBA–MDA adducts changes with temperature. The fluorescence intensity of the TBA–MDA adduct was observed to decrease with increasing temperature. Specifically, as the temperature rose from 20 °C to 60 °C, the fluorescence intensity diminished by threefold (Figure 1E,F). This decline in fluorescence activity, coupled with unaltered UV-vis extinction (Figure S5), suggests a decrease in fluorescence quantum yield with increasing temperature [26]. This is because quantum yield is related to the ratio of emitted photons to absorbed photons [27]. Reduced fluorescence quantum yield with increasing temperature has been commonly reported, with several factors believed to decrease fluorophore quantum yield at elevated temperatures, including external conversion, internal conversion, and intersystem crossing, significantly contributing to the reduction in fluorescence intensities [27,28].

However, the efficiency of the kinetic CG assay is significantly influenced by temperature variations (Figure 1G,H). Generally, at elevated temperatures, the rate of the TBA–MDA reaction increases. When the temperature is increased from 20 °C to 60 °C, the time required to reach the end of the linear region for CG quantification significantly decreases, resulting in more than a fivefold improvement in assay efficiency. The expedited reaction at elevated temperatures led to improved overall kinetic CG assay efficiency, with a kinetic quantification time of 7 min, 4.5 min, 3.18 min, 1.91 min, and 1.2 min at 20 °C, 30 °C, 40 °C, 50 °C, and 60 °C, respectively (Figure 1H).

### 3.2. Calibration, Precision, and Recovery

**Figure 2:**
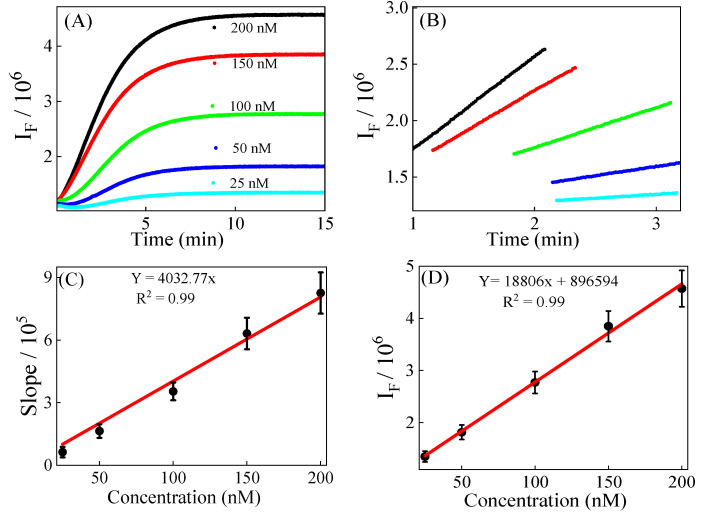
(**A**) The kinetic fluorescence of the reaction solutions of MDA standards and 205.2 mM TBA in DMSO/water cosolvents. The concentration of MDA standards in the reaction solution is 25 nM, 50 nM, 100 nM, 150 nM, and 200 nM, respectively. (**B**) The linear curve-fitting of the kinetic fluorescence from 1 to 4 min. (**C**) The linear calibration curve between the (dots) slope of the kinetic data and the MDA concentration. (**D**) The linear calibration curve between the end point of the spectra and concentration. The kinetic fluorescence was excited at 532 nm and detected at 567 nm; the slit widths of the excitation and detection monochromators are 3 nm and 28 nm, respectively.

External standard calibration curves (Figure 2A) were established for both the kinetic and equilibrium methods by plotting analyte concentration (x-axis) against the corresponding signal (y-axis). The curves were generated using standard MDA solutions ranging from 25 nM to 200 nM, after reaction with 205.2 mM TBA in a 50% DMSO/water mixture at 60 °C. The resulting linear regression equations were y = 4032.77x for the kinetic method and y = 18,806x + 896,594 for the equilibrium method. The kinetic method is more sensitive than the equilibrium method, as evidenced by its LOD (0.87 nM) and LOQ (2.6 nM) compared to the equilibrium method (LOD 2.28 nM and LOQ 6.8 nM). The equilibrium approach exhibits slightly better intra-day (0.30% vs. 0.59%) and inter-day precision (2.56% vs. 2.84%) because it measures a fully developed, stable signal after reaction completion, reducing variability. Although the kinetic method shows a slightly higher RSD%, both methods demonstrate excellent precision with values well within acceptable analytical limits. Furthermore, a recovery experiment was conducted using the analysis of beef samples spiked with 0 nM, 40 nM, and 80 nM MDA solution, respectively. The recovery percentage was higher in the equilibrium method (95.2–124.5%) compared to the kinetic method (95.0–104.7%). These results can be explained as the equilibrium method is known to often give higher calculated MDA concentrations than the actual MDA concentration because the probe has enough time to react with both MDA and matrix components, resulting in a higher detected signal. By contrast, the kinetic method measures MDA concentration during the reaction, which reduces the chance of interference from the sample matrix. Table 1 presents the relevant analytical parameters discussed above.

Kinetic quantification is based on the slope of the kinetic spectroscopic data in the linear time course region. So, the kinetic calibration curve is a plot of the slope of the signals in the linear time course region as a function of time. As shown in Figure 2B, this linear region is achieved between 1 to 4 min, indicating that the overall efficiency of the kinetic quantification process is less than 4 min.

The calibration curve for equilibrium quantification is based on the final data obtained after the reaction reaches equilibrium. The calibration curve is a plot of fluorescence intensity over concentration. In our case, we collected a data point at 15 min to construct the calibration curve. We opted to collect the 15-min data point because, at 205.2 mM concentration of TBA, the TBA–MDA reaction reaches equilibrium by 15 min when no interfering matrices are present (Figure 2A).

Siu and Draper [29] reported that the time to reach equilibrium for the TBA–MDA reaction using water as solvent took 90 min at 80 °C. Similarly, Leon [14] reported that it took two hours for the completion of the TBA–MDA reaction at 95 °C. This indicates that the overall efficiency of the equilibrium method using water for the TBA–MDA reaction ranges from 90 to 120 min, making it at least 15 times slower than the optimized kinetic method (typical efficiency 6 min).

### 3.3. Method Validation

The identical R^2^ values (0.99) for the calibration curves of both the kinetic and equilibrium methods (Figure 2C,D) suggest that the kinetic and equilibrium methods are equally reliable, provided there are no matrix interferences. However, the primary challenge when applying spectroscopic methods in real-world scenarios is the vulnerability to matrix interferences.

The crucial question is how the kinetic method compares to the equilibrium method in systems containing complex matrices. To answer this question, the kinetic method was validated by using sample volume variation and standard addition methods for ground beef samples. To mitigate these matrix interferences, an extraordinarily low *v*/*v*% extract of ground beef (3.8% or lower extract of ground beef) was added to the reaction solution.

**Figure 3:**
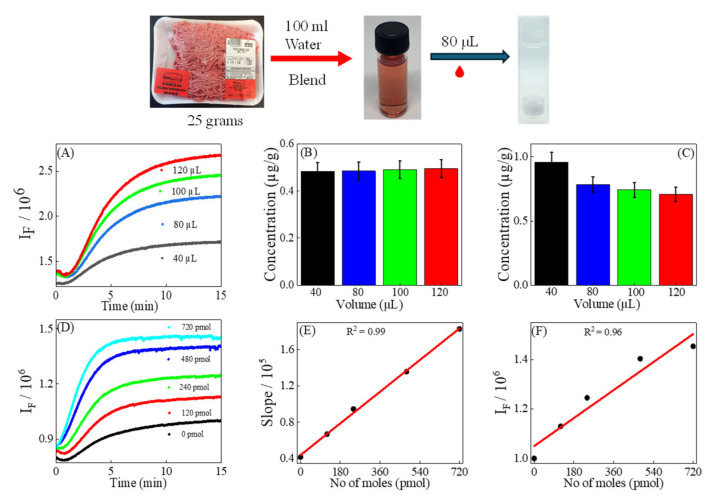
(**A**) Kinetic fluorescence of reaction solutions of 205 mM TBA mixed with different volumes of the extract of ground beef, as shown in the legend. (**B**,**C**) Predicted MDA concentration using the external calibration curve from the kinetic and equilibrium quantification methods. (**D**) Kinetic fluorescence of the reaction solutions of TBA and extracts of ground beef with different amounts of added MDA standards. (**E**,**F**) Linear curve-fitting for standard-addition quantification of MDA concentration in the extract of ground beef using kinetic and equilibrium methods.

In this study, FG quantification of MDA in four distinct reaction solutions, varying only in volume (40 µL, 80 µL, 100 µL, and 120 µL) of the extract of ground beef (prepared as detailed in Section 2.5) was added to reaction solutions containing 2.44 mL of 205.2 mM TBA in 50% DMSO/water and 0.5 mL of 1.3 M H_3_PO_4_. The data was collected after a reaction time of 3.5 min, then the MDA concentration of each solution was calculated using the calibration curve and appropriate dilution factors. The average MDA concentration for these samples using kinetic quantification was found to be 0.48 ± 0.05 µg/g of ground beef (Figure 3B). This very low standard deviation indicates the kinetic method has a remarkable reproducibility, showing no significant matrix interferences.

The average MDA concentration in the same samples, determined using the equilibrium method, was found to be 0.79 ± 0.11 µg/g of ground beef. As the volume of beef extract increases, the calculated MDA concentration decreases (Figure 3C), resulting in a much higher standard deviation than the kinetic method. The reduction in MDA concentration with higher volumes of extract of ground beef is likely due to matrix interferences affecting the fluorescence baselines. The fluorescence baselines impact equilibrium quantification but do not affect kinetic assays, as kinetic measurements rely on the change in fluorescence intensity during the reaction. Nonetheless, the amounts of MDA in the ground beef deduced from external standard calibration using both kinetic and equilibrium fluorescence methods are less than the acceptance threshold for rancid off flavors as previously reported by Campo et al. [5].

In this sample volume variation experiment, the *t*-test produced a *t*-statistic of −5.383 with a *p*-value of 0.0062. The absolute value of this *t*-statistic surpassed the critical *t*-value of 3.18. This indicates that the MDA concentration calculated using the equilibrium method is significantly different from concentrations calculated by the kinetic method at a confidence level of 94%.

The method was further validated using the standard addition method. The excellent linearity (R^2^ = 0.99) between the slopes of kinetic fluorescence spectra from solutions differing only in the amount of MDA standard (Figure 3E) added to each solution indicates the effectiveness and the robustness of the kinetic fluorogenic method. The concentration of MDA calculated for the extract of ground beef used for the standard-added solutions is 39.03 ± 2.73 nM. The equilibrium method was also examined using the standard addition method. The data reveal no strong linearity between the fluorescence intensities and the amount of added MDA standard to the extract of ground beef solution. The weak relationship is likely due to the presence of the sample inner filter effect observed at high MDA concentration [30]. The results from sample volume variation and standard addition indicate that the kinetic method is more reliable than the equilibrium method in complex systems with matrix interferences.

The successful kinetic fluorescence quantification of MDA in the extract of ground beef demonstrated in this work and in the canned chicken used in the earlier work demonstrated that with adequate sample dilution, matrix interference in practical samples can be mitigated to a negligible contribution. Given that MDA concentrations in biological samples, such as meats and human urine, typically range from sub- to low-µM range [31,32] which are well above the sub-nanomolar detection limit of the kinetic fluorescence method, a dilution factor of 100 or more can be used without compromising MDA detectability using either standard addition or external standard calibrations.

### 3.4. Quantification of MDA in Ground Beef Exposed to Air

**Figure 4:**
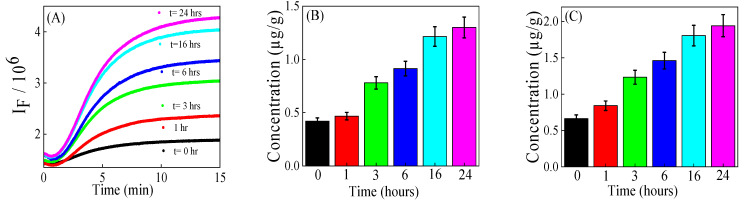
(**A**) Kinetic fluorescence of reaction solutions of 205 mM TBA mixed with 80 µL of extract of ground beef exposed to air at different time intervals as shown in the legend. (**B**) MDA concentration using kinetic fluorescence of TBA–MDA reaction. (**C**) MDA concentration using the equilibrium fluorescence of TBA–MDA reaction.

The concentration of MDA in ground beef exposed to ambient air was determined using the kinetic FG method (Figure 4). This was achieved by exposing approximately 25 g of ground beef (*n* = 3) in an open atmosphere for different time intervals from 0 to 24 h. As expected, the concentration of MDA in ground beef increased from 0.41 ± 0.02 to 1.29 ± 0.11 µg/g after being exposed to ambient air over a 24-h period. The concentration of MDA increased by a factor of nearly 3.14 after 24 h. Raines and Hunt [33] reported that the MDA concentration in ground beef patties exposed for 0 to 48 h ranged from 2.49 to 5.03 µg/. The trends are similar, but the higher MDA content in this previous study compared to the current study can be attributed to three main differences in experimental parameters (previous vs. current): the higher oxygen percentage (80% O_2_ vs. 21% O_2_), the variation in exposure time (48 h vs. 24 h), and the fat composition in the ground beef (50% fat vs. 27% fat).

The equilibrium method also determined that the MDA level significantly increased, rising from 0.56 ± 0.05 to 1.94 ± 0.15 µg/g after 24 h of air exposure. The MDA concentration (1.94 µg/g) after 24 h is 1.49 times higher compared to the kinetic method (1.29 µg/g). The elevated MDA levels observed with the equilibrium method can be partly attributed to TBARS-reactive substances having longer time to react with TBA, thereby enhancing signal generation or interference from matrix fluorescence background.

In the air-exposed experiment, the *t*-test yielded a *t*-statistic of −7.79 (*p* = 0.0002), whose absolute value exceeded the critical *t*-value of 2.015. This indicates a significant difference in the MDA concentrations measured by the two methods. In the air-exposed experiment, the 95% confidence interval for the kinetic method was (0.463–1.234 µg/g), while the equilibrium method showed a wider interval of (0.786–1.861 µg/g), both calculated with a degree of freedom (df) of 5. The Bland–Altman plot is given in Figure S6.

The increased level of MDA in ground beef exposed to air for longer time intervals is consistent with literature reports [34,35,36]. The rise in MDA concentration with longer exposure time is partly attributed to the increased opportunity for oxygen to interact with polyunsaturated fatty acids [37,38]. These results support the advice to consumers to avoid long-term exposure of ground beef to an open atmosphere.

### 3.5. Quantification of MDA in Extracts of Washed Beef

**Figure 5:**
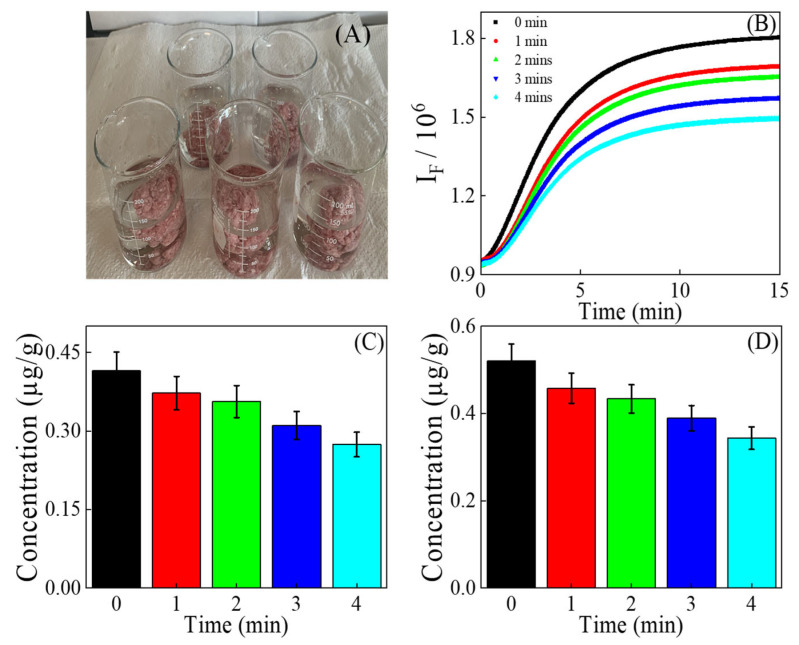
(**A**) Photograph of ground beef washed in water. (**B**) Kinetic fluorescence of the reaction solutions of TBA and extracts of ground beef washed for different time intervals. (**C**) MDA concentration using kinetic fluorescence quantification. (**D**) MDA concentration using equilibrium fluorescence quantification.

Motivated by MDA’s solubility in water, we conducted a washing experiment to determine how much MDA could be removed from ground beef (Figure 5). The ground beef was washed by soaking in water with continuous stirring using a stir bar for 0 to 4 min. After decanting the water, the ground beef was blended to prepare extract of washed ground beef. The MDA concentration, as measured by the kinetic fluorescence method, decreased with longer washing times. After 4 min of washing, it dropped by a factor of 1.48, reaching 0.27 ± 0.01 µg/g (Figure 5C). A similar result was observed using the equilibrium quantification method, though the factor of MDA reduction was higher, around 1.51. The higher reduction of MDA concentration in washed beef using the equilibrium method is partly attributed to the removal of TBARS-reactive substances along with MDA during the washing process or the high intercept in the equilibrium calibration curve. To our knowledge, this is the first experiment conducted on ground beef to investigate the impact of washing on MDA content. The finding indicates that consumers should be advised to wash ground beef before cooking to reduce their intake of MDA. The calculated statistics were 13.60 (*p* = 0.00017), whose absolute value exceeded the critical *t*-value of 2.13. This suggests that there is a significant difference in the MDA concentration calculated from kinetic and equilibrium methods. During the washing experiment, the confidence interval for the kinetic method was narrower (0.277–0.413 µg/g) than that for the equilibrium method (0.345–0.512 µg/g), with a degree of freedom of 4. The Bland–Altman plot for washed ground beef is given in Figure S6.

### 3.6. Quantification of MDA in Cooked Ground Beef

**Figure 6:**
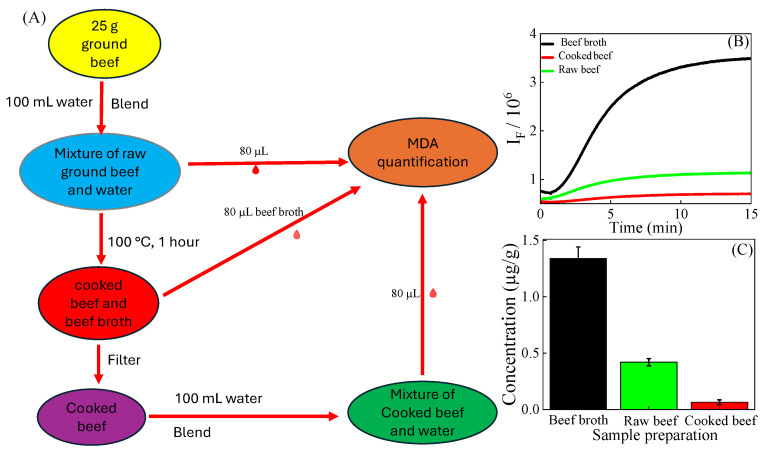
(**A**) Scheme for quantification of MDA. (**B**) Kinetic FG reaction of TBA with extracts of ground beef obtained with different processing methods. (**C**) MDA concentration using the kinetic quantification method.

Since the dawn of human civilization, cooking meat has been a common practice. Cooking enhances the aroma, flavor, and tenderness of meat compared to its raw state. However, cooking can affect the nutritional value of meat and sometimes leads to an increase in harmful components [39,40,41]. In our study, we determined the MDA concentration in raw ground beef (raw beef) and cooked ground beef (cooked beef) along with its broth (beef broth) (Figure 6B,C). We discovered that the concentration of MDA in ground beef broth increased by a factor of 3.19 after cooking, reaching a level of 1.34 ± 0.10 µg/g.

There is little research on ground beef boiled in water. Siu and Draper [29] reported that the concentration of MDA in boiled ground beef broth is 1.89 µg/g. The difference in MDA concentration between this previous study and the current study can be attributed to differences (previous vs. current) in cooking temperature (80 °C vs. 100 °C), cooking time (90 min vs. 60 min), and the varying composition of the ground beef used (unknown vs. 27% fat). An increase in MDA levels after cooking meat is commonly reported in the literature [42]. The increase in MDA content in cooked ground beef is due to the increased availability of iron and oxygen, which promotes lipid peroxidation [43,44].

Interestingly, we discovered that the MDA content in cooked ground beef was found to be 0.23 ± 0.01 µg/g. This level is lower than that in both the raw ground beef and the beef broth, by factors of 1.78 and 5.82, respectively. The result provides an important finding that boiling meat in water, followed by decantation of the liquid before eating, is beneficial.

## 4. Conclusions

This work provides, for the first time, vital information on the impact of solvent and temperature on CG and FG equilibrium and kinetic MDA assays. Among the solvents explored, a (1:1, *v*/*v*) DMSO/water cosolvent offered the best combination of high sensitivity and efficiency. Using the optimal solvent and temperature found in this study, MDA quantification using the kinetic FG assay had an efficiency of 4–6 min, which is at least fifteenfold faster than MDA equilibrium-based assays used in commercially available kits (60–90 min). Sample volume variation was highly effective in mitigating matrix interferences in kinetic assays. We also found that the MDA content in ground beef increases with both exposure to ambient air and cooking. However, both washing the ground beef and decanting cooked ground beef broth reduce MDA levels in the ground beef. To promote safer ground beef handling and consumption practices, consumers are advised to wash the meat thoroughly for a few minutes, then boil it and discard the liquid before eating.

## Figures and Tables

**Table 1:** Analytical parameters of the kinetic and equilibrium methods.

Analysis Method	LOD (nM)	LOQ (nM)	R^2^	Intra-Day Precision	Inter-Day Precision	Recovery Percentage
Kinetic	0.87	2.61	0.99	0.59%	2.84%	95.00–104.70%
Equilibrium	2.28	6.84	0.99	0.30%	2.56%	95.24–124.50%

## Data Availability

The original contributions presented in this study are included in the article/Supplementary Materials. Further inquiries can be directed to the corresponding author(s).

## Supplementary Materials

The following supporting information can be downloaded at: https://www.mdpi.com/article/10.3390/foods14142525/s1. Text S1: Python program with graphic user interface, Figure S2: Linear region identified by using Savitzky-Golay derivatization, Figure S3: Solvent dependent UV-vis spectra of TBA-MDA adduct, Figure S4: Kinetic fluorescence reaction of 50 nM standard MDA with 500 mM TBA solution prepared in DMSO/water cosolvent and 100 mM of TBA in water, Figure S5: Temperature dependent UV-vis spectra of TBAMDA adduct and Figure S6: Bland-Altman plots for the ground beef exposed to air and washed ground beef.

## Abbreviations

The following abbreviations are used in this manuscript:

MDA	malondialdehyde
TBA	2-thiobarbituric acid
CG	chromogenic
FG	fluorogenic
TBARS	TBA-reactive substances
HPLC	high-performance liquid chromatography
TEP	tetraethoxypropane
SERS	surface-enhanced Raman spectroscopic
DMSO	dimethyl sulfoxide
DMF	dimethylformamide
LOD	limit of detection
LOQ	limit of quantification
RSD	relative standard deviation
