# Supplementary material for: Rapid Kinetic Fluorogenic Quantification of Malondialdehyde in Ground Beef
Source: Foods. 2025 Jul 18;14(14):2525. doi: 10.3390/foods14142525 (PMC12295034; doi:10.3390/foods14142525)
Supplement: Supplementary file 1 [file foods-14-02525-s001.zip › foods-3735609-supplementary.pdf]

foods

MDPI

Rapid Kinetic Fluorogenic Quantification of Malondialdehyde in Ground Beef

Keshav Raj Bhandari [1], *Max Wamsley* [1], *Bindu Nanduri* [2], *Willard E. Collier* [3,*], *Dongmao Zhang* [1,*]

[1] Department of Chemistry, Mississippi State University, Mississippi State, MS 39762, United States

[2] Department of Basic sciences, Mississippi State University, Mississippi State, MS 39762, United States

[3] Department of Chemistry, Tuskegee University, Tuskegee, AL 36088, United States

*Willard E. Collier; wcollier@tuskegee.edu; (662)-861-9630

*Dongmao Zhang; dongmao@chemistry.msstate.edu; (662)-325-6752

Table of Contents

S1. Python program with graphic user interface……………………………………………………………..3

S2. Linear region identified by using Savitzky-Golay derivatization……………………………………6

S3. Solvent dependent UV-vis spectra of TBA/MDAadduct……………………………………….......7

S4. Kinetic fluorescence reaction of 50 nM standard MDA with 500 mM TBA solution prepared in DMSO/water cosolvent and 100 mM of TBA in water ………………………………………………8

S5. Temperature dependent UV-vis spectra of TBA/MDA adduct………...9

S6. Bland-Altman plots for the ground beef exposed to air and washed ground beef….....................10

Text S1. Python program with graphic user interface

Python Graphic User Interface program for taking Savitzky-Golay derivative. It allows users to upload Excel files containing data and compute the first derivative of the second column (Y) with respect to the first column (X) using the Savitzky-Golay filtering method. The user can try different window sizes for taking Savitzky-Golay derivative

```
import tkinter as tk
from tkinter import ttk, filedialog, messagebox
import pandas as pd
import numpy as np
from scipy.signal import savgol_filter
import matplotlib.pyplot as plt
from matplotlib.backends.backend_tkagg import FigureCanvasTkAgg

class DerivativeApp:
    def __init__(self, root):
        self.root = root
        self.root.title("Savitzky-Golay Derivative from Excel")

        # Upload Excel button
        ttk.Button(root, text="Upload Excel File (.xlsx)", command=self.load_excel).pack(pady=10)

        # Window size input
        ttk.Label(root, text="Window size (odd number ≥ 3):").pack()
        self.window_entry = tk.Entry(root)
        self.window_entry.pack()

        # Derivative button
        ttk.Button(root, text="Compute Derivative", command=self.compute_derivative).pack(pady=10)

        # Plot area
        self.figure = plt.Figure(figsize=(6, 4))
        self.ax = self.figure.add_subplot(111)
        self.canvas = FigureCanvasTkAgg(self.figure, master=root)
        self.canvas.get_tk_widget().pack()

        # Data holders
        self.x = None
        self.y = None

    def load_excel(self):
        file_path = filedialog.askopenfilename(filetypes=[("Excel files", "*.xlsx")])
```

```
        if file_path:
            try:
                df = pd.read_excel(file_path)
                if df.shape[1] < 2:
                    raise ValueError("Excel must have at least two columns.")
                self.x = df.iloc[:, 0].values
                self.y = df.iloc[:, 1].values

                # Plot raw data immediately
               self.ax.clear()
                self.ax.plot(self.x, self.y, label='Original Data')
                self.ax.set_title("Uploaded Data (X vs Y)")
                self.ax.legend()
                self.canvas.draw()

                messagebox.showinfo("Success", "Excel data loaded and plotted.")
            except Exception as e:
                messagebox.showerror("Error", f"Failed to load Excel:\n{e}")

    def compute_derivative(self):
        try:
            if self.x is None or self.y is None:
                raise ValueError("No data loaded. Upload an Excel file first.")
            window_size = int(self.window_entry.get())
            if window_size % 2 == 0 or window_size < 3:
                raise ValueError("Window size must be an odd number ≥ 3.")
            if window_size > len(self.x):
                raise ValueError("Window size cannot exceed number of data points.")

            dy_dx = savgol_filter(self.y, window_length=window_size, polyorder=2, deriv=1, delta=(self.x[1] - self.x[0]))

            # Plot original + derivative
           self.ax.clear()
            self.ax.plot(self.x, self.y, label='Original Data')
            self.ax.plot(self.x, dy_dx, label='1st Derivative', linestyle='--')
            self.ax.set_title("Savitzky-Golay First Derivative")
            self.ax.legend()
            self.canvas.draw()

        except Exception as e:
            messagebox.showerror("Error", str(e))

# Run the app
```

```
if __name__ == "__main__":
    root = tk.Tk()
    app = DerivativeApp(root)
    root.mainloop()
```

S2. Linear region identified by using Savitzky-Golay derivatization

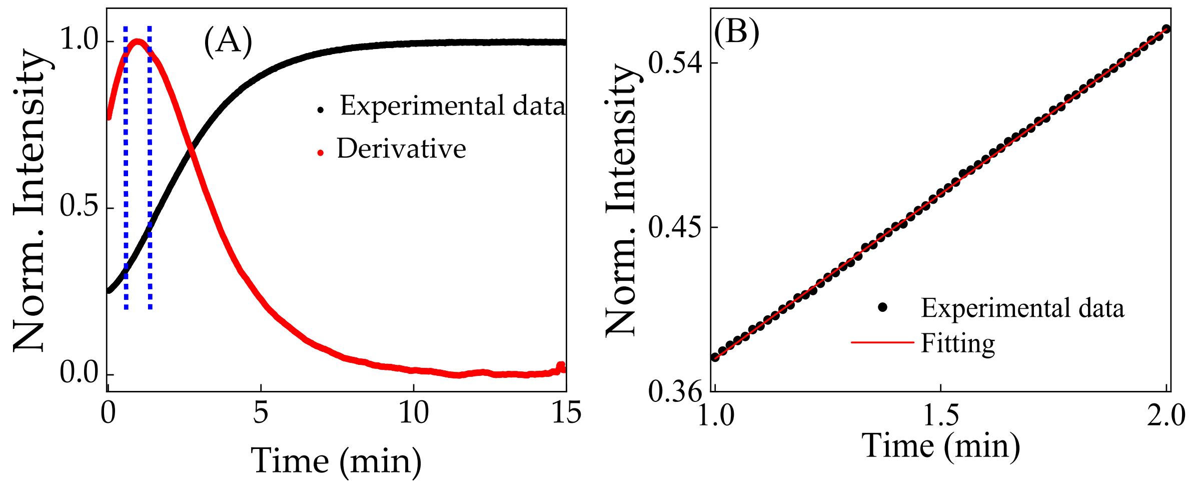

Figure S2: Fig: (A) Kinetic fluorescence reaction of 200 nM standard MDA with 205.2 mM TBA as a function of the reaction time. The black line is experimental data, and the red lines are the first derivative obtained with second order Savitzky-Golay method with window size of 11. (B) Linear curve-fitting of the kinetic spectral intensity in the linear time-courses. The linear time course region where the rate of the spectral intensity change is within 5% difference from its maximum.

S3. Solvent dependent UV-vis spectra of TBA-MDA adduct

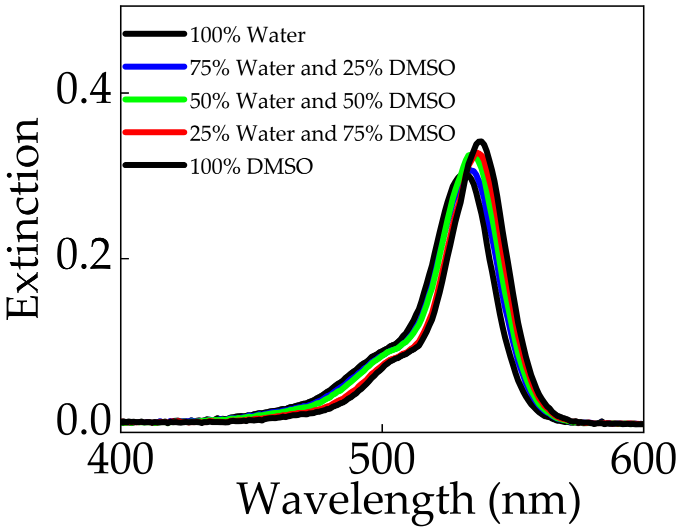

Figure S3. Solvent dependent UV-vis extinction spectra of 1 μM TBA-MDA adduct in DMSO/water. cosolvents of different DMSO volume fractions.

S4. Kinetic fluorescence reaction of 50 nM standard MDA with 500 mM TBA solution prepared in DMSO/water cosolvent and 100 mM of TBA in water.

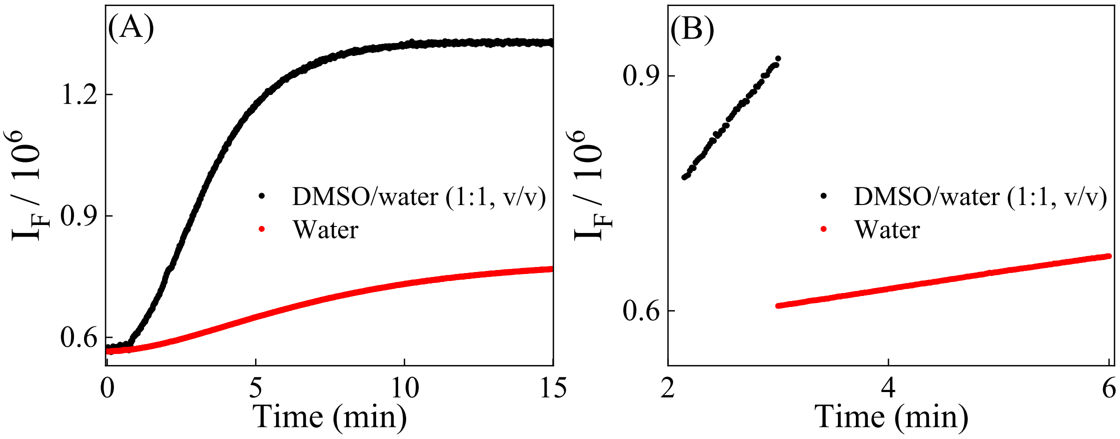

Figure S4: (A) Kinetic fluorescence reaction of a 50 nM standard MDA solution at 60°C, with a 500 mM TBA solution prepared in a DMSO/water cosolvent, and 100 mM of TBA in water, as a function of reaction time. (B) Linear time course region obtained for TBA-MDA reaction in (1:1, v/v) DMSO/water cosolvent and water.

S5. Temperature dependent UV-vis spectra of TBA/MDA adduct.

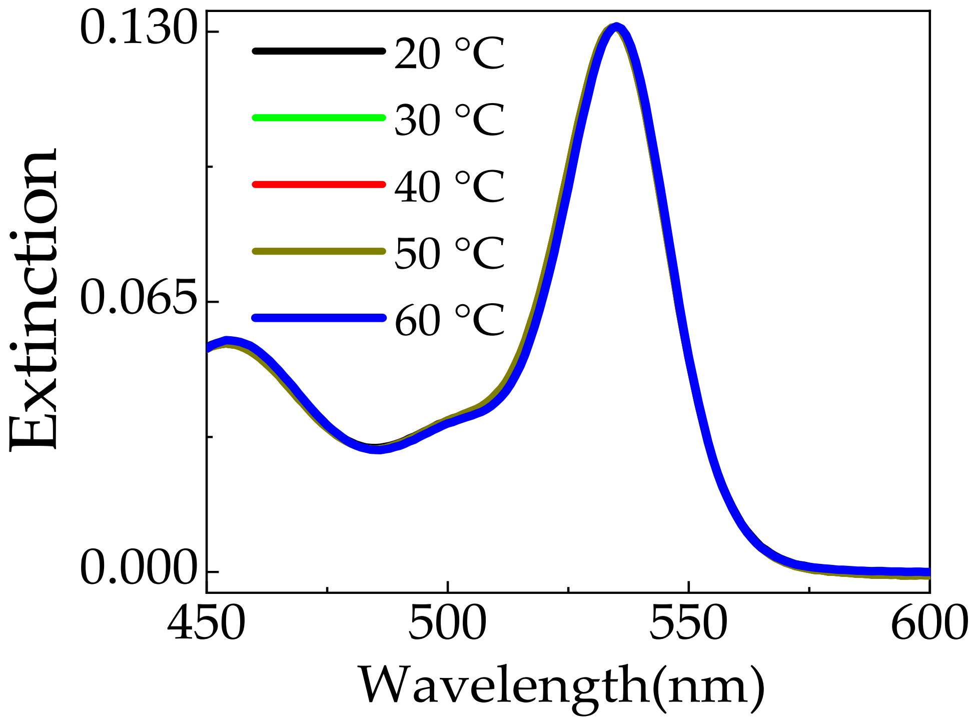

Figure S5: Temperature dependent UV-vis extinction spectra of 1 μM TBA-MDA adduct in (1:1, v/v) DMSO/water cosolvent fraction.

S6. **Bland-Altman plots for the ground beef exposed to air and washed ground beef**

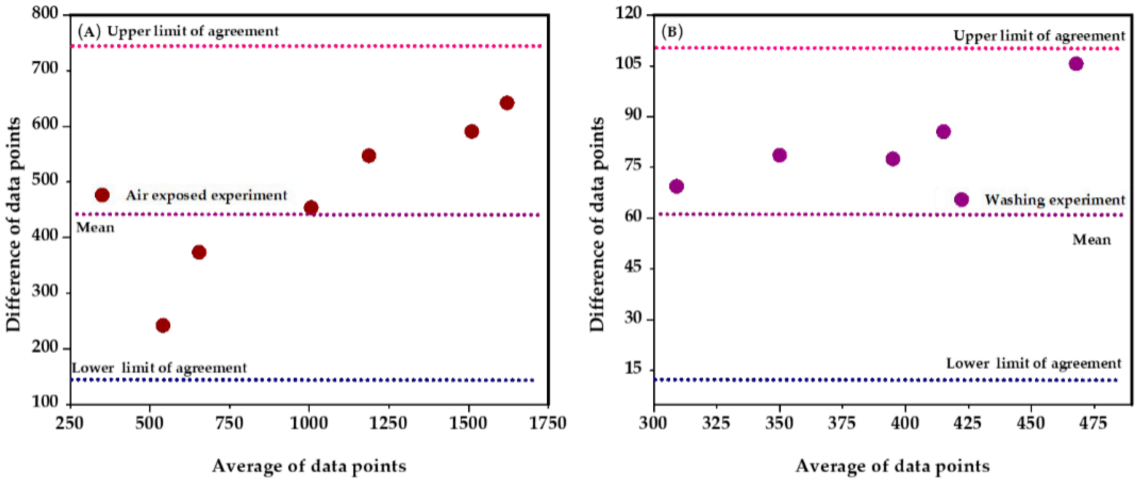

Figure S6: (A) Bland-Altman plot for the ground beef exposed to air. (B) Bland-Altman plot for washed ground beef.
